# The American Gynecological Society

**Published:** 1887-10

**Authors:** 


### *THE AMERICAN GYNECOLOGICAL SOCIETY.*

The twelfth annual meeting of the American Gynecological Society, held in the Academy of Medicine, New York, on the 13th, 14th and 15th days of September, 1887, will long be remembered as a most notable gathering of medical men. Following, as it did, so close upon upon the International Medical Congress, held in Washington the previous week, it naturally attracted the attendance of the most distinguished gynecologists who were at the Congress, and who had been especially invited to attend this meeting in New York.

Among the noted foreign visitors were: Dr. August Martin, of Berlin; Dr. A. Cordes, of Geneva, Switzerland; Dr. George Granville Bantock and Dr. Grailly Hewitt, of London; Dr. Lawrence, of Bristol, Eng.; Dr. Apostoli, Dr. Terrier and Dr. Doléris, of Paris; Dr. Alexander R. Simpson, of Edinburgh; Dr. George H. Kidd, of Dublin; Dr. Balls-Headley, of Melbourne, Australia; and Dr. P. G. Unna, of Hamburg. Drs. Bantock, Doléris and Apostoli read papers, and most of the others took part in the discussions.

The Society was fortunate in having for its president at this time, Dr. Alexander J. C. Skeene, of Brooklyn, a genial gentleman and an accomplished presiding officer, under whose guidance the work was so expedited that the entire programme, a long one, was finished at the appointed time.

To Dr. Fordyce Barker was assigned the pleasing duty of welcoming the Society and its guests to the hospitalities of the occasion. Fit man, fit task. No one to-day can be more truly said to be a representative of American medicine than Fordyce Barker. His years sit kindly on his graceful physique, and his fluent, finished, classic English is always delightsome to the ear. The scientific work done at this meeting was of an exceptionally good quality, embracing, besides those of the foreign members already mentioned, papers by Drs. Emmet, Busey, Polk, Mundé, Palmer, Hunter, Jackson, Chadwick, Battey, Johnstone, Parvin, Van de Werker, Foster, and Jewett, together with the annual address of the president, which was of more than ordinary interest.

The social part of the entertainment included a reception by Dr. Fordyce Barker on Tuesday evening, luncheons at Delmonico's on Tuesday, Wednesday and Thursday, at one o'clock P. M., and the annual dinner of the Society, also at Delmonico's, on Thursday evening, at seven o'clock. The luncheon of Wednesday was given by the New York Obstetrical Society, upon its special card of invitation. The members and invited guests, both American and foreign, were present for the most part on these several occasions. The mid-day recess, from one to three o'clock, passed in such pleasant companionship, served to promote acquaintance and strengthen friendships, which cannot prove otherwise than beneficial to all the fortunate participants of these bounteous hospitalities.

In addition to the regular programme, an opportunity was afforded several of the gentlemen present to witness laparotomies, Alexander's operation, etc., at Bellevue hospital, through the courtesies of the operators, Dr. W. Gill Wylie and Dr. Wm. M. Polk.

The Society did itself honor in selecting Dr. Robert Battey, of Rome, Ga., as the president for the ensuing year, a guaranty of efficient and conscientious continuation of good work. It negotiated the proposition to become a part of the American Congress of Physicians and Surgeons. For the successful carrying out of an elaborate schedule of great scientific and social interest and value, too much praise cannot be awarded Drs. Mundé and Lee, the indefatigable executive committee, whose great glory must be the memory of the meeting of '87.

