# Correction

**Published:** 1887-11

**Authors:** 


CORRECTION.—In our editorial on the American Gynecological Society in the October number, the proof-reviser made us say "negotiated," whereas we wrote "negatived," in speaking of th e society's action upon the proposition to join the congress of special societies. This society rejected the offer.

